# Hispidin Ameliorates Acute Ultraviolet B-Induced Skin Inflammation by Targeting Reactive Oxygen Species-Dependent Neutrophil Extracellular Trap Formation

**DOI:** 10.3390/ijms27083667

**Published:** 2026-04-20

**Authors:** Yuina Arakaki, Koshi Tominaga, Keiichi Hiramoto, Masashi Imai, Akihiro Morita, Tomonari Tsutsumi, Hiroyuki Yasuda, Eisuke F. Sato

**Affiliations:** 1 Department of Biochemistry, Faculty of Pharmaceutical Sciences, Suzuka University of Medical Science, Suzuka-City 513-8670, Mie, Japan; yuina88.tyappy@icloud.com (Y.A.); guranareguria53@gmail.com (K.T.); hiramoto@suzuka-u.ac.jp (K.H.); dp23001@st.suzuka-u.ac.jp (M.I.); morita-a@suzuka-u.ac.jp (A.M.); tutumi@suzuka-u.ac.jp (T.T.); 2 Division of Pathological Sciences, Department of Pharmacology and Experimental Therapeutics, Kyoto Pharmaceutical University, Misasagi 5, Yamashina, Kyoto 607-8414, Kyoto Prefecture, Japan; yasuda20@mb.kyoto-phu.ac.jp

**Keywords:** neutrophil extracellular trap, NETosis, hispidin, UVB, skin inflammation, NLRP3, peptidylarginine deiminase 4

## Abstract

Excessive neutrophil extracellular trap (NET) formation (NETosis), frequently associated with reactive oxygen species (ROS), exacerbates cutaneous inflammation induced by acute ultraviolet B (UVB) exposure. Although hispidin has potent antioxidant activity, its protective effects against acute UVB-induced skin inflammation and its relationship with NET-associated responses remain unclear. We investigated the effects of topical hispidin on acute UVB-induced skin injury in mice and examined its effects on ROS-associated NET-related responses in differentiated HL-60 cells. In a mouse model, topical hispidin (0.1% and 0.5%) ameliorated UVB-induced skin damage in a dose-dependent manner, as evidenced by improved clinical and histological findings. Hispidin treatment was associated with reduced systemic oxidative stress and decreased cutaneous expression of CXCL2, C5a, IL-1β, NLRP3, Ly6G, PAD4, and citrullinated histone H3. In differentiated HL-60 cells, hispidin reduced ROS-associated signals and suppressed PMA-triggered extracellular DNA release, but did not suppress A23187-triggered extracellular DNA release under experimental conditions. Cell viability analysis showed that hispidin did not significantly affect differentiated HL-60 cell viability at tested concentrations under the present experimental conditions. Topical hispidin alleviates acute UVB-induced skin inflammation by suppressing neutrophil infiltration and NET-related inflammatory responses. Hispidin may therefore represent a promising candidate as a topical modulator of oxidative stress- and NET-associated skin inflammation.

## 1. Introduction

Ultraviolet (UV) radiation from sunlight is broadly classified into UVA, UVB, and UVC based on wavelength. Among these, UVB reaches the Earth’s surface and exerts potent biological effects on skin. Acute exposure to UVB induces rapid inflammatory responses that manifest as erythema, edema, and pain through direct phototoxic injury to epidermal cells and activation of inflammatory signaling pathways [1,2]. In contrast, chronic and cumulative UVB exposure contributes to photoaging and photocarcinogenesis by promoting extracellular matrix degradation, cellular senescence, DNA photolesions and dysregulation of key signaling pathways [3,4]. Therefore, elucidating the mechanisms underlying UVB-induced skin injury and identifying effective preventive strategies remain important issues in dermatology research.

Among the inflammatory processes induced by UVB, increasing attention has been directed toward the contribution of innate immune cells, particularly neutrophils. Neutrophils are rapidly recruited to sites of tissue injury, where they perform several effector functions, including the formation of neutrophil extracellular traps (NETs). NETs are extracellular web-like structures composed of decondensed chromatin decorated with histones and granular proteins that can immobilize and eliminate pathogens [5]. However, excessive or dysregulated NET formation (NETosis) contributes to tissue injury and has been implicated in a wide range of inflammatory and autoimmune disorders [6]. Recent studies have shown that UVB irradiation induces marked neutrophil infiltration and NET-related responses in murine skin, suggesting that NETosis may contribute to the amplification of acute UVB-induced skin inflammation [7].

Although the molecular mechanisms governing NET-associated inflammatory responses remain incompletely understood, reactive oxygen species (ROS) are widely recognized as important upstream regulators of NETosis. ROS generated by NADPH oxidase (NOX) promote chromatin decondensation and NET release through signaling events involving PAD4-mediated histone citrullination [8,9]. Thus, antioxidant compounds may be promising candidates for suppressing NET-associated inflammatory injury [10].

*Alpinia zerumbet*, commonly known as Gettou, is a perennial plant native to Southeast Asia and Okinawa, Japan, and has long been used in food and traditional medicine. Phytochemical studies have shown that A. zerumbet contains bioactive compounds with antioxidant, anti-inflammatory, and antimicrobial activities [11]. Hispidin, a polyphenolic compound found in A. zerumbet, has been reported to possess antioxidant and cytoprotective activities in several experimental settings [12]. However, its effects on acute UVB-induced skin inflammation and its relationship with neutrophil infiltration and NET-associated responses remain unclear.

In this study, we investigated whether topical hispidin ameliorates acute UVB-induced skin injury in mice and examined its effects on ROS-associated NET-related responses in differentiated HL-60 cells. Because our in vivo working model assumes that UVB first induces tissue injury and inflammatory signaling in the skin, followed by neutrophil recruitment into the inflamed lesion, the in vitro experiments were designed as pathway-oriented assays rather than direct reconstruction of UVB exposure to neutrophils. In addition, because the stimulus responsiveness and functional properties of HL-60-derived neutrophil-like cells depend on the differentiation protocol [13,14,15], retinoic acid-differentiated cells were used for PMA stimulation, whereas DMSO-differentiated cells were used for A23187 stimulation. Using this experimental framework, we evaluated whether the topical and cellular effects of hispidin are associated with the attenuation of oxidative stress, neutrophil recruitment, and NET-related inflammatory responses in acute UVB-induced skin inflammation.

## 2. Results

### 2.1. Topical Hispidin Ameliorates UVB-Induced Clinical and Histological Skin Damage

**Figure 1:**
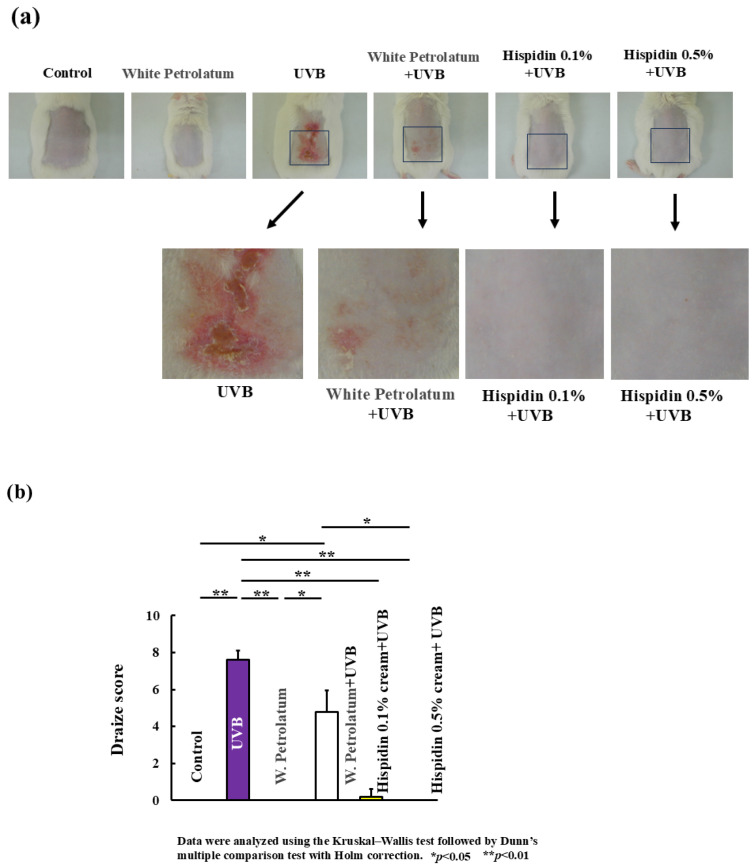
Topical hispidin ameliorates UVB-induced skin damage in mice. (**a**) Representative photographs of the dorsal skin from the indicated experimental groups on day 8. Mice were exposed to UVB irradiation (1.0 kJ/m^2^/day) for 3 consecutive days and treated topically with vehicle (white Vaseline) or hispidin cream (0.1% or 0.5%) once daily for 8 consecutive days. (**b**) Clinical severity of dermatitis was evaluated on day 8 using the modified Draize scoring system. The total score (0–8) was calculated as the sum of the erythema (0–4) and edema (0–4) scores. Data are presented as median with interquartile range (*n* = 6 per group). Statistical significance was analyzed using the Kruskal–Wallis test, followed by Dunn’s multiple comparison test. * *p* < 0.05, ** *p* < 0.01 versus the UVB + vehicle group. UVB, ultraviolet B.

To evaluate the in vivo effects of hispidin, we first examined whether the topical application of hispidin affected the clinical appearance of UVB-irradiated mouse skin. As shown in Figure 1a, UVB exposure induced marked skin lesions, including erythema, edema, and scaling in the UVB + vehicle group. In contrast, topical treatment with hispidin (0.1% or 0.5%) visibly improved UVB-induced skin changes. The severity of dermatitis was quantified using a modified Draize scoring system (Figure 1b). Consistent with the representative photographs, the UVB + vehicle group showed increased clinical scores, whereas both concentrations of topical hispidin reduced the severity of the clinical phenotype. This effect was more pronounced in the 0.5% hispidin group.

**Figure 2:**
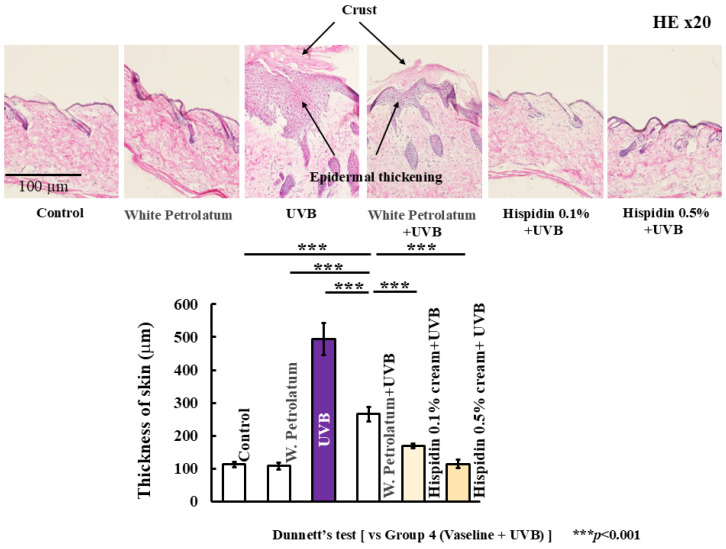
Topical hispidin reduces UVB-induced epidermal thickening. Representative hematoxylin and eosin-stained sections of the dorsal skin and quantitative analysis of epidermal thickness in the indicated experimental groups. Scale bar = 100 μm. Data are presented as mean ± SD (*n* = 6 per group). Statistical significance was analyzed using one-way ANOVA, followed by Dunnett’s multiple comparison test. *** *p* < 0.001 versus the UVB + vehicle group. UVB, ultraviolet B.

To determine whether these gross findings were accompanied by histological improvement, we examined hematoxylin and eosin-stained skin sections (Figure 2). UVB irradiation induced marked epidermal thickening and inflammatory cell infiltration in the dorsal skin, whereas topical hispidin reduced these histological changes. These results indicate that topical hispidin ameliorates both clinical and histological manifestations of acute UVB-induced skin injury.

### 2.2. Topical Hispidin Reduces Systemic Oxidative Stress and Cutaneous IL-1β Expression

**Figure 3:**
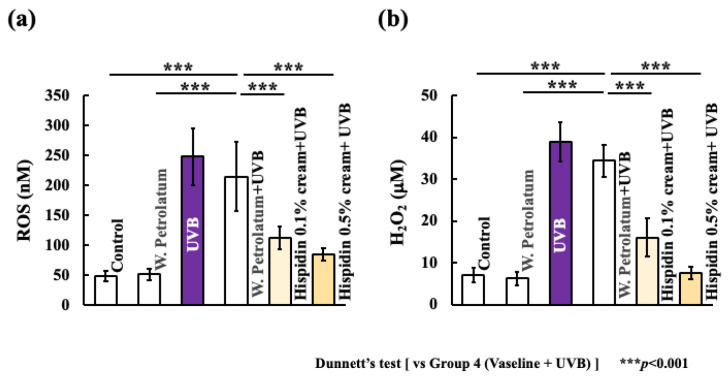
Topical hispidin reduces systemic oxidative stress after UVB irradiation. (**a**) Quantitative determination of serum ROS-associated signals in the indicated experimental groups. (**b**) Quantitative determination of serum H_2_O_2_ levels in the indicated experimental groups. Data are presented as mean ± SD (*n* = 6 per group). Statistical significance was analyzed using one-way ANOVA, followed by Dunnett’s multiple comparison test. *** *p* < 0.001 versus the UVB + vehicle group. UVB, ultraviolet B.

**Figure 4:**
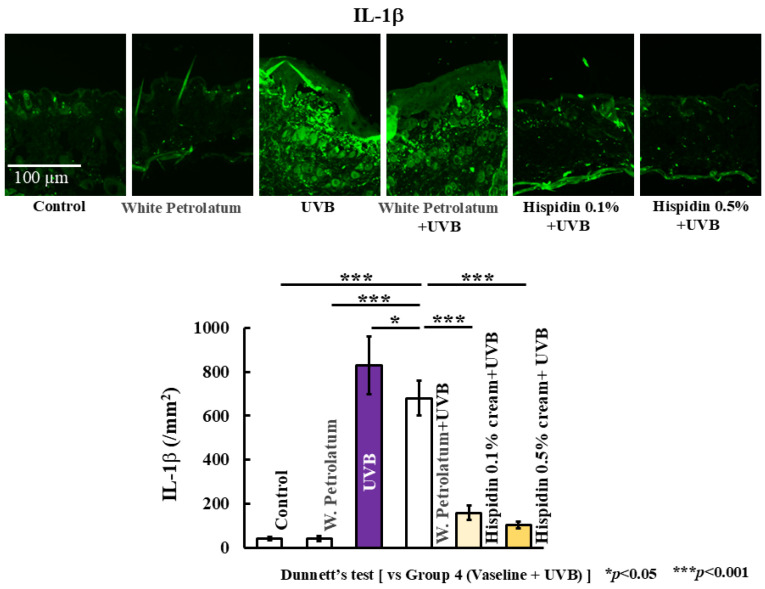
Topical hispidin suppresses IL-1β expression in UVB-irradiated skin. Representative immunohistochemical or immunofluorescence images of IL-1β in dorsal skin sections and quantitative analysis of IL-1β-positive cells in the indicated experimental groups. Scale bar = 100 μm. Data are presented as mean ± SD (*n* = 6 per group). Statistical significance was analyzed using one-way ANOVA, followed by Dunnett’s multiple comparison test. * *p* < 0.05, *** *p* < 0.001 versus the UVB + vehicle group. UVB, ultraviolet B.

Because oxidative stress is a central feature of acute UVB-induced tissue injury, we next examined systemic oxidative stress markers. As shown in Figure 3, UVB irradiation increased the levels of serum oxidative stress markers compared to those in the non-irradiated control group, whereas topical hispidin treatment reduced these elevations. The suppressive effect was greater in the 0.5% hispidin group than that in the 0.1% group. We then assessed the expression of the pro-inflammatory cytokine IL-1β in the dorsal skin tissue. Strong IL-1β staining was observed in UVB-irradiated skin, whereas this signal was attenuated by topical hispidin treatment (Figure 4). Again, this reduction was more evident in the 0.5% hispidin group. These findings indicate that the protective effects of topical hispidin are associated with the attenuation of both systemic oxidative stress and local inflammatory cytokine expression.

### 2.3. Topical Hispidin Suppresses Neutrophil Chemoattractant Expression and Reduces NET-Related Markers in UVB-Irradiated Skin

**Figure 5:**
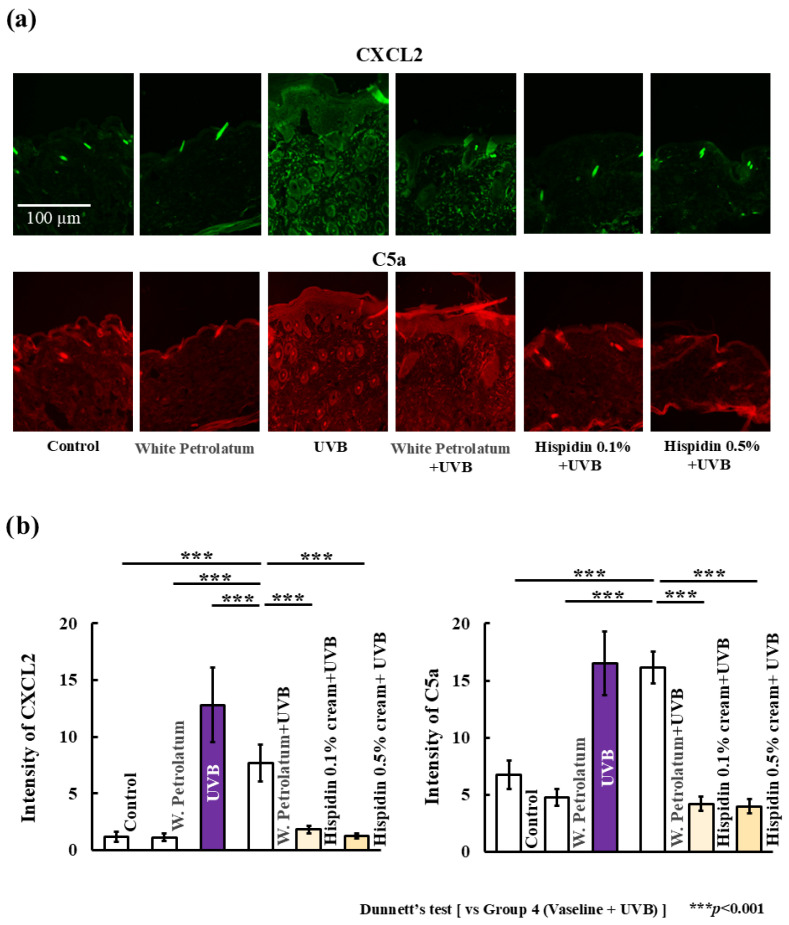
Topical hispidin suppresses neutrophil chemoattractant expression in UVB-irradiated skin. (**a**) Representative images of CXCL2 and C5a expression in dorsal skin sections from the indicated experimental groups. (**b**) Quantitative analysis of CXCL2 and C5a expression. Scale bar = 100 μm. Data are presented as mean ± SD (*n* = 6 per group). Statistical significance was analyzed using one-way ANOVA followed by Dunnett’s multiple comparison test. *** *p* < 0.001 versus the UVB + vehicle group. UVB, ultraviolet B.

To explore the mechanism underlying the reduced inflammatory cell infiltration, we examined the expression of neutrophil chemoattractants in UVB-irradiated skin. As shown in Figure 5, UVB exposure increased the expression of CXCL2 and C5a, whereas topical hispidin treatment reduced the expression of both mediators. This reduction was observed at both concentrations and was more pronounced in the 0.5% hispidin group.

**Figure 6:**
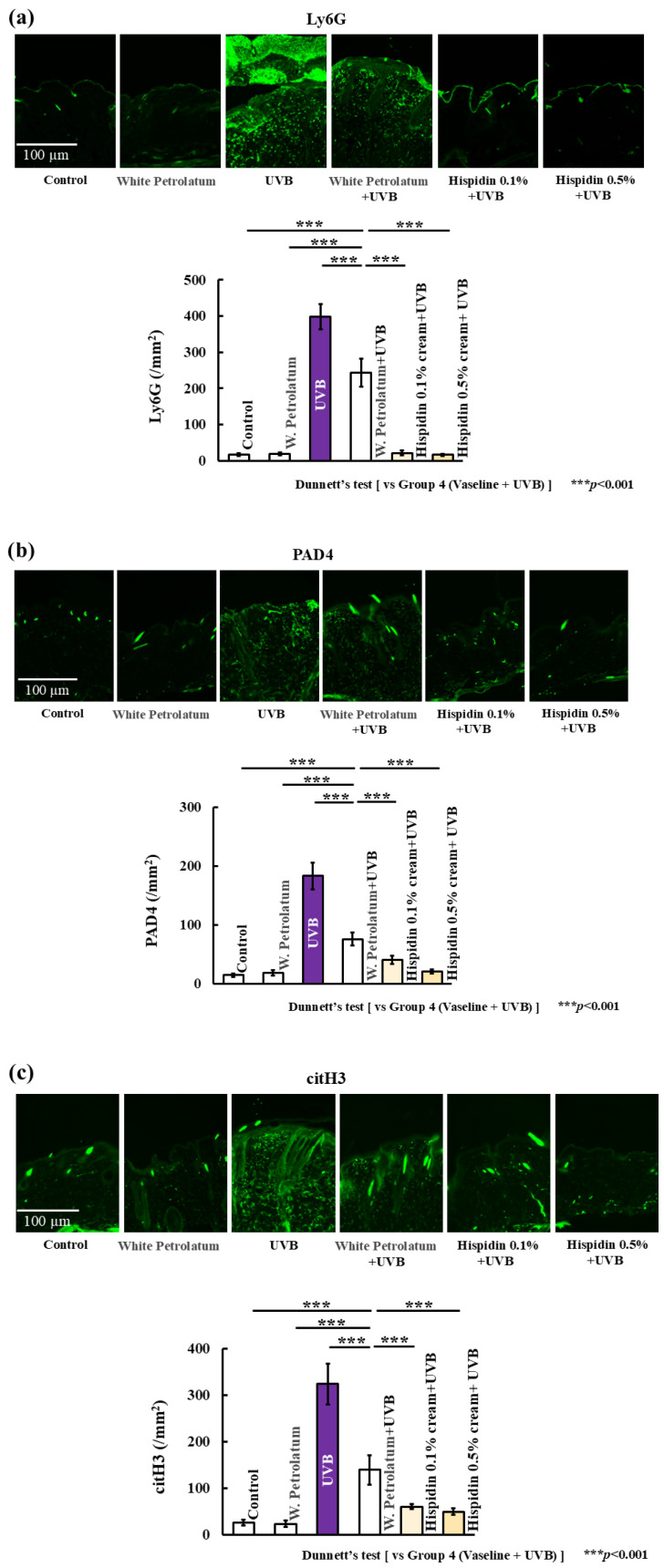
Topical hispidin reduces neutrophil infiltration and NET-related markers in UVB-irradiated skin. (**a**) Representative images and quantitative analysis of Ly6G-positive neutrophils in dorsal skin sections. (**b**) Representative images and quantitative analysis of PAD4-positive cells. (**c**) Representative images and quantitative analysis of citrullinated histone H3 (citH3)-positive cells. Scale bar = 100 μm. Data are presented as mean ± SD (*n* = 6 per group). Statistical significance was analyzed using one-way ANOVA, followed by Dunnett’s multiple comparison test. *** *p* < 0.001 versus the UVB + vehicle group. UVB, ultraviolet B.

Next, we examined neutrophil accumulation and NET-related markers in the skin tissue. UVB irradiation markedly increased the number of Ly6G-positive cells and enhanced the expression of PAD4 and citrullinated histone H3 (citH3) (Figure 6). In contrast, topical hispidin treatment reduced Ly6G-positive cell infiltration and lowered the expression of PAD4 and citH3. These results indicate that topical hispidin is associated with reduced neutrophil recruitment and lower expression of NET-related markers in UVB-irradiated skin.

### 2.4. Topical Hispidin Suppresses NLRP3 Expression in UVB-Irradiated Skin

**Figure 7:**
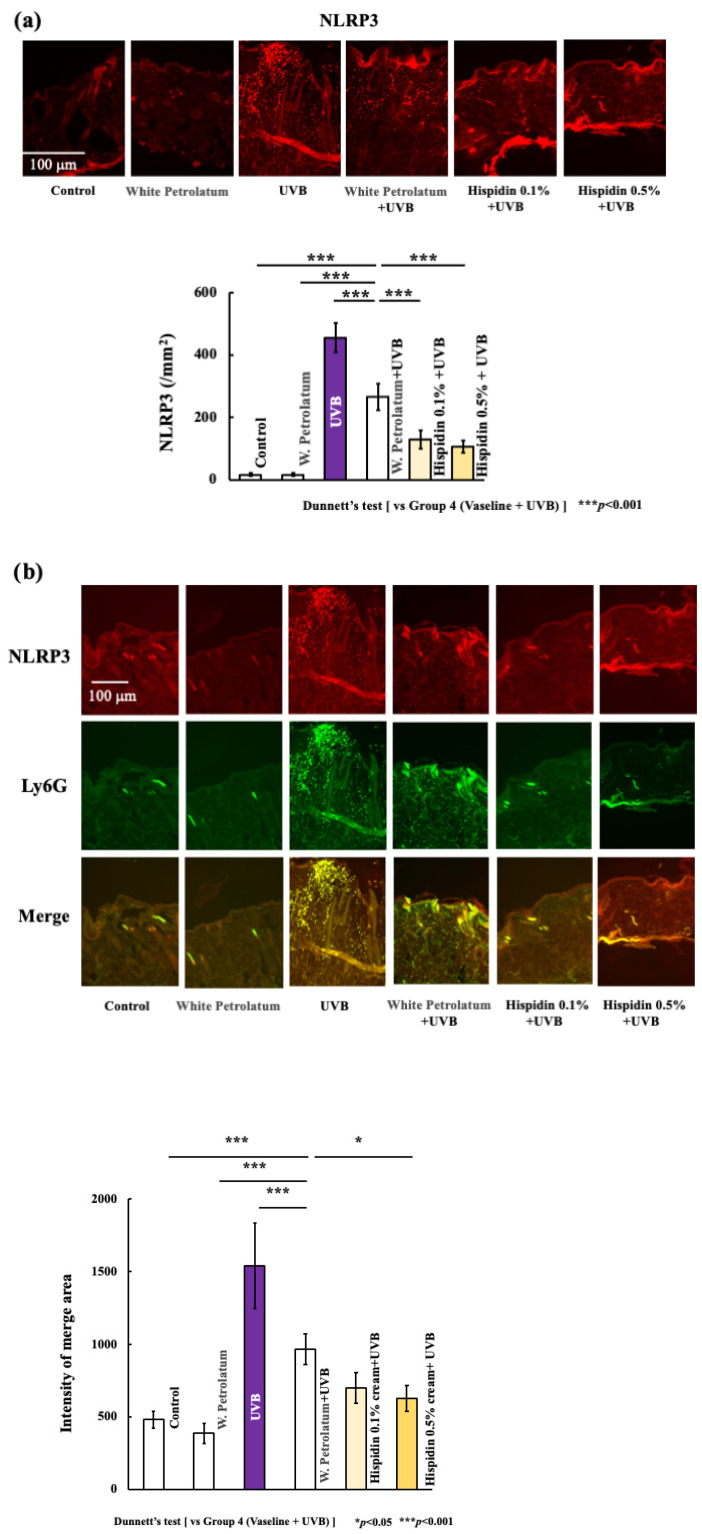
Topical hispidin suppresses NLRP3 expression in UVB-irradiated skin. (**a**) Representative images and quantitative analysis of NLRP3-positive cells in dorsal skin sections from the indicated experimental groups. (**b**) Representative immunofluorescence images showing NLRP3, Ly6G, and merged images in the indicated groups. Scale bar = 100 μm. Data are presented as mean ± SD (*n* = 6 per group). Statistical significance was analyzed using one-way ANOVA followed by Dunnett’s multiple comparison test. * *p* < 0.05, *** *p* < 0.001 versus the UVB + vehicle group. UVB, ultraviolet B.

Because IL-1β production and NET-related responses are closely linked to inflammasome-associated signaling, we next examined NLRP3 expression in the skin. As shown in Figure 7a, UVB irradiation markedly increased the number of NLRP3-positive cells in the dorsal skin, whereas topical hispidin treatment reduced this increase in a dose-dependent manner. In addition, immunofluorescence analysis showed that NLRP3 signals were predominantly localized in association with Ly6G-positive cells in inflamed lesions (Figure 7b). This finding suggests that infiltrating neutrophils substantially contribute to NLRP3-associated responses in acute UVB-induced inflammatory settings. Together, these results indicate that topical hispidin is associated with the suppression of NLRP3 expression in UVB-irradiated skin.

### 2.5. Hispidin Suppresses ROS-Associated NET-Related Responses in Differentiated HL-60 Cells

**Figure 8:**
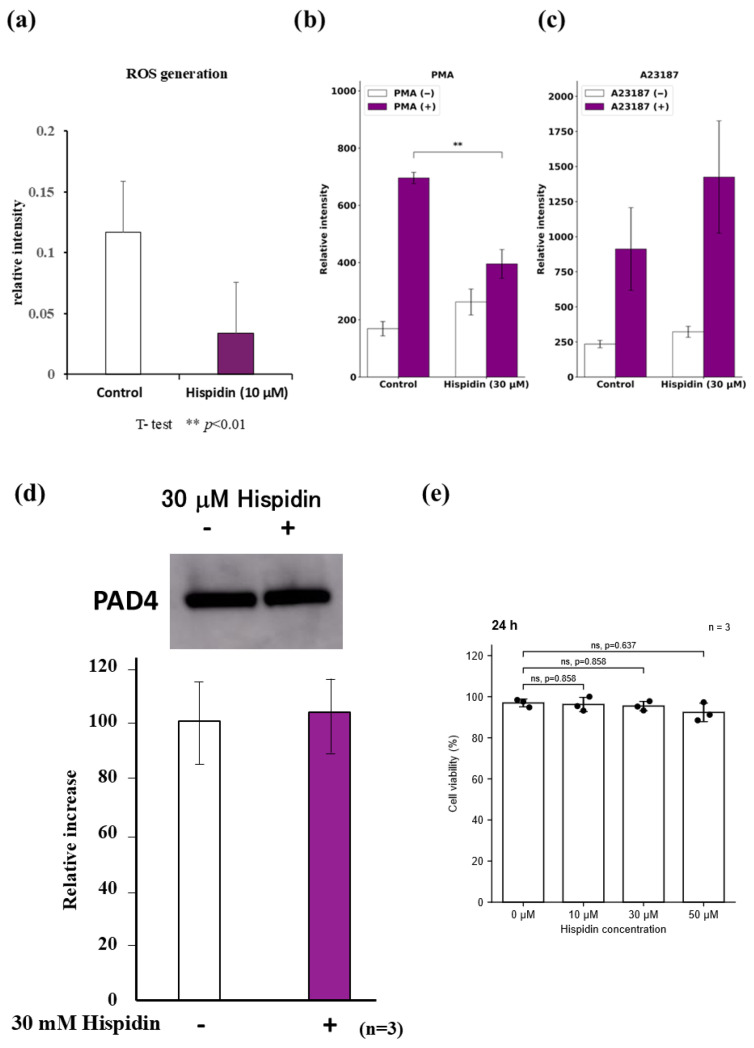
Effects of hispidin on ROS-associated NET-related responses in differentiated HL-60 cells. (**a**) Intracellular ROS generation after treatment with hispidin (10 μM) was evaluated in differentiated HL-60 cells. ** *p* < 0.01 (**b**) Effect of hispidin (30 μM) on PMA-triggered extracellular DNA release. (**c**) Effect of hispidin (30 μM) on A23187-triggered extracellular DNA release. (**d**) PAD4 protein expression after treatment with hispidin (30 μM). A representative immunoblot is shown; the experiment was performed independently three times. (**e**) Effects of hispidin on dHL-60 cell viability after 24 h treatment with the indicated concentrations. Bars show mean ± SD and dots indicate independent replicates (*n* = 3). Statistical significance for panel (**e**) was analyzed using Holm-adjusted Welch *t*-tests versus the 0 μM control. NETosis, neutrophil extracellular trap formation; PMA, phorbol 12-myristate 13-acetate.

To further examine whether hispidin affects neutrophil-related responses under in vitro conditions, we used differentiated HL-60 (dHL-60) cells. As shown in Figure 8a, hispidin reduced intracellular ROS-associated signals in dHL-60 cells. Next, we examined the release of extracellular DNA in response to two established stimuli. Hispidin reduced PMA-triggered extracellular DNA release (Figure 8b) but did not suppress A23187-triggered extracellular DNA release under the present experimental conditions (Figure 8c). To further explore whether hispidin alters PAD4 expression, we performed Western blot analysis. As shown in Figure 8d, hispidin did not markedly alter PAD4 protein expression in dHL-60 cells. Taken together, these findings indicate that under the present experimental conditions, hispidin suppressed PMA-triggered ROS-associated NET-related responses without markedly altering PAD4 expression. Therefore, the PMA and A23187 assays should be interpreted as pathway-oriented experiments rather than strict one-to-one quantitative comparisons.

### 2.6. Effects of Hispidin on dHL-60 Cell Viability

To assess whether the effects of hispidin on ROS-associated NET-related responses were influenced by cytotoxicity, we examined the viability of differentiated HL-60 cells after treatment with hispidin. As shown in Figure 8e, hispidin did not significantly affect cell viability at 10, 30, or 50 μM after 24 h treatment under the present experimental conditions. Similar results were also observed after 48 h treatment (Figure S1). These findings indicate that the inhibitory effects observed in the in vitro assays are unlikely to be explained simply by nonspecific cytotoxicity.

## 3. Discussion

The present study provides in vivo evidence that topical hispidin ameliorates acute UVB-induced skin injury and is associated with the suppression of NET-related inflammatory responses. Although compounds derived from Alpinia zerumbet have long been recognized for their antioxidant and anti-inflammatory properties [11,12], the present findings extend this knowledge by demonstrating that topical application of hispidin reduces UVB-induced skin inflammation, oxidative stress, neutrophil infiltration, and NET-related markers in vivo. In addition, our in vitro experiments support the hypothesis that hispidin suppresses ROS-associated neutrophil responses under specific experimental conditions.

Our in vivo findings suggest that the protective effects of hispidin are linked to the attenuation of the inflammatory cascade triggered by UVB irradiation. Topical hispidin reduced clinical skin damage and epidermal thickening and was associated with lower systemic oxidative stress markers. These changes were accompanied by reduced cutaneous expression of the neutrophil chemoattractants CXCL2 and C5a, decreased accumulation of Ly6G-positive neutrophils, and lower levels of PAD4 and citrullinated histone H3. Collectively, these findings support the interpretation that hispidin attenuates UVB-induced skin inflammation, at least in part, by reducing oxidative stress and subsequent neutrophil-driven inflammatory amplification. This interpretation is consistent with previous studies showing that UVB exposure is associated with neutrophil infiltration and NET-related responses in murine skin [7], and with the broader view that ROS are important upstream regulators of NETosis [8,9,10].

The reduction in IL-1β and NLRP3 expression is consistent with the suppression of the inflammatory environment in UVB-exposed skin. The critical involvement of the NLRP3 inflammasome in UVB-induced skin inflammation has been previously reported [16,17,18,19]. In the present model, NLRP3 signals were predominantly associated with Ly6G-positive cells, suggesting that infiltrating neutrophils contribute substantially to inflammasome-related responses in this acute inflammatory setting. Therefore, the observed decrease in neutrophil recruitment after hispidin treatment may explain the reduced expression of NLRP3, IL-1β, and NET-related markers in vivo. Rather than proving a single linear pathway, our data support a model in which hispidin mitigates multiple interconnected inflammatory events downstream of UVB-induced tissue injury.

To further explore the cellular mechanisms, we used differentiated HL-60 cells as an in vitro model. In these assays, hispidin reduced ROS-associated signals and suppressed PMA-triggered extracellular DNA release. In contrast, it did not suppress A23187-triggered extracellular DNA release under the present conditions and did not markedly alter PAD4 protein expression. These findings are consistent with the possibility that hispidin preferentially affects ROS-associated signaling rather than PAD4 expression [8,9,10]. However, these in vitro data should be interpreted with caution. The PMA and A23187 experiments were not intended as a strict one-to-one quantitative comparison. Instead, they were designed as pathway-oriented assays under stimulus-appropriate conditions. This approach is supported by previous studies showing that functional responsiveness in HL-60-derived neutrophil-like cells depends on the differentiation protocol and may differ according to the stimuli used [13,14].

An important point in the present study is that the in vitro experiments were not designed to model direct UVB irradiation of neutrophils. Our working hypothesis in vivo is that UVB first induces tissue injury and inflammatory signaling in the skin, followed by the recruitment of neutrophils into the inflamed lesion, where NET-related responses occur [7]. From this pathophysiological perspective, we used PMA and A23187 as pathway-oriented stimuli to probe ROS-associated and ROS-independent responses, rather than directly reconstructing UVB exposure. Because stimulus responsiveness and functional properties of HL-60-derived neutrophil-like cells depend on the differentiation protocol [13,14,15], RA-differentiated cells were used for PMA stimulation, whereas DMSO-differentiated cells were used for A23187 stimulation. Accordingly, these assays were performed under stimulus-appropriate conditions, and the results should be interpreted in that context rather than as a strict side-by-side comparison between the two stimuli.

The cell viability data further support the interpretation of the in vitro findings. Hispidin did not significantly affect dHL-60 cell viability at the tested concentrations after 24 h treatment, and similar results were also observed after 48 h treatment. Therefore, the suppression of ROS-associated signals and PMA-triggered extracellular DNA release is unlikely to be attributable to nonspecific cytotoxicity. Nevertheless, broader validation using additional in vitro NETosis-related markers and matched experimental conditions would further strengthen this mechanistic interpretation.

This study had several limitations. First, no benchmark inhibitor was included, which limits the direct comparison of the magnitude of the effect of hispidin with that of established anti-inflammatory or NETosis-modulating agents [10]. Second, the in vivo study was performed using a mouse model, and the mechanistic assays relied on differentiated HL-60 cells rather than primary human neutrophils. Third, some endpoints, including Draize scoring, histology, and image quantification, were assessed without blinding, which should be acknowledged as a methodological limitation. Finally, although the data are consistent with suppression of ROS-associated NET-related responses, the precise molecular target of hispidin remains unclear. Future studies should include benchmark compounds, broader in vitro marker validation, primary human neutrophils, and experimental systems that more closely model the inflammatory microenvironment generated by UVB-damaged skin.

Overall, the present data support a working model in which topical hispidin alleviates acute UVB-induced skin inflammation through combined attenuation of oxidative stress, inflammatory mediator expression, neutrophil recruitment, and NET-related responses. While additional mechanistic validation is warranted, these findings identify hispidin as a promising candidate for further investigation as a topical modulator of oxidative stress- and NET-associated skin inflammation.

## 4. Materials and Methods

### 4.1. Animals

All animal experiments were conducted in accordance with the animal care regulations of Suzuka University of Medical Science (Suzuka, Mie, Japan) and were approved by the Animal Experiment Ethics Committee of Suzuka University of Medical Science (approval number: 31; approved on 25 September 2014). Specific pathogen-free, 8-week-old male ICR mice (Japan SLC Co., Hamamatsu, Japan) were used in the experiments. Under light sevoflurane anesthesia, the dorsal fur of the mice was shaved using an electric clipper. Mice were randomly assigned to the experimental groups. No animals were excluded from the analysis. The investigators were not blinded to the group allocation during the experiment.

### 4.2. Induction of UVB Irradiation-Induced Skin Damage

The whole body of lightly anesthetized mice was exposed to UVB irradiation (wavelength range: 280–320 nm, peak at 305 nm) using a sunlamp (FL-20SE; Toshiba Co., Tokyo, Japan) for 3 consecutive days at a dose of 1.0 kJ/m^2^ per day (45 s/day). Other wavelengths were filtered using a Kodaul cellulose film (Eastman Kodak Co., Rochester, NY, USA). A dorsal skin area of 2 × 4 cm (8 cm^2^) was shaved on each mouse. Mice were divided into the following six groups: Control, White Vaseline, UVB irradiation, UVB irradiation + White Vaseline, UVB irradiation + 0.1% hispidin cream, and UVB irradiation + 0.5% hispidin cream.

Hispidin (0.1% or 0.5%; Tokyo Chemical Industry Co., Ltd., Tokyo, Japan) was formulated in white Vaseline (Kenei Pharmaceutical Co., Osaka, Japan) as the vehicle. The selected concentrations were used as practical exploratory topical doses to evaluate dose dependency within a formulation range that could be reproducibly prepared and stably applied in vivo. White Vaseline or hispidin cream was applied once daily for 8 consecutive days. Neither hispidin nor Vaseline was applied during UVB irradiation; instead, the treatment was applied after irradiation. The daily application dose was 16 mg per 8 cm^2^ site, corresponding to 2 mg/cm^2^. On day 8, photographs of the dorsal skin were taken, and blood and skin samples were collected for further analyses.

### 4.3. Draize Scoring System

To evaluate the severity of dermatitis after UVB irradiation, the dorsal skin was assessed for erythema and edema using a modified Draize scoring system [20]. Total scores ranged from 0 to 8 and were calculated as the sum of the erythema score (0–4) and edema score (0–4). Erythema was graded as follows: 0, no erythema; 1, very slight erythema; 2, well-defined erythema; 3, moderate-to-severe erythema; and 4, severe erythema. Edema was graded as follows: 0, no edema; 1, very slight edema; 2, slight edema; 3, moderate edema; and 4, severe edema extending beyond the area of exposure.

### 4.4. Preparation and Staining of Dorsal Skin Sections

On the final day of the experiment, skin samples were collected under anesthesia. Dorsal skin specimens were fixed in 4% phosphate-buffered paraformaldehyde, embedded in Tissue-Tek OCT compound (Sakura Finetek, Tokyo, Japan), and cut into 5 μm thick sections. The sections were stained with hematoxylin and eosin for histological evaluation [21]. For immunohistological analyses [22], sections were incubated with the following primary antibodies: mouse monoclonal anti-Ly6G (1:100; BD Biosciences, Franklin Lakes, NJ, USA), rabbit polyclonal anti-citrullinated histone H3 (citH3; 1:100; Abcam, Cambridge, MA, USA), rabbit polyclonal anti-PAD4 (1:100; Abcam), rabbit monoclonal anti-CXCL2 (1:100; ab317569, Abcam), rabbit polyclonal anti-C5a (1:100; bs-0324R; Bioss Antibodies, Woburn, MA, USA), rabbit polyclonal anti-IL-1β (1:100; ab205924, Abcam), and anti-NLRP3 (1:100; AG-20B-0014-C100, AdipoGen, San Diego, CA, USA). After washing, the sections were incubated with fluorescein isothiocyanate-conjugated anti-mouse or anti-rabbit secondary antibodies (1:30; Dako Cytomation, Glostrup, Denmark). Images were obtained using a fluorescence microscope. Quantification of Ly6G, PAD4, citH3, IL-1β, CXCL2, C5a, and NLRP3 signals was performed using ImageJ software ver. 1.53 (National Institutes of Health, Bethesda, MD, USA) from four randomly selected fields of a constant area.

### 4.5. Quantification of Systemic Oxidative Stress

Serum oxidative stress was evaluated using the OxiSelect In Vitro ROS/RNS Assay Kit (Green Fluorescence) (Cell Biolabs, San Diego, CA, USA). Samples were added to black 96-well plates along with hydrogen peroxide standards to generate a standard curve. Fluorescence intensity was measured using a SpectraMax plate reader (485 nm excitation, 525 nm emission; Molecular Devices Japan, Tokyo, Japan). The results were expressed according to the manufacturer’s instructions.

### 4.6. Cell Culture

HL-60, a human promyelocytic leukemia cell line (RCB3683; RIKEN BioResource Center, Ibaraki, Japan), was cultured in RPMI 1640 medium (Nacalai, Kyoto, Japan) supplemented with 10% (*v*/*v*) heat-inactivated fetal bovine serum and 1% penicillin/streptomycin (Fujifilm Wako, Osaka, Japan) [23]. Cells were maintained at 37 °C in a humidified incubator with 5% CO_2_, and the culture medium was replaced every 2 days. To induce neutrophil-like differentiation, HL-60 cells were cultured with 1.25% DMSO for 3 days [24]. Alternatively, the cells were differentiated using 1 μM retinoic acid (RA). Because stimulus responsiveness in HL-60-derived neutrophil-like cells depends on the differentiation protocol [13,14,15], RA-differentiated cells were used for PMA stimulation, whereas DMSO-differentiated cells were used for A23187 stimulation. Hispidin was added after differentiation, and cells were incubated with the indicated concentrations for 24 h before analysis.

### 4.7. Cell Viability Assay

Cell viability was assessed to evaluate the concentration-dependent effects of hispidin on the differentiated HL-60 cells. After differentiation, the cells were treated with the indicated concentrations of hispidin for 24 h or 48 h under the same conditions used for the mechanistic assays. The cell suspension was then mixed 1:1 with 0.4% trypan blue solution in microtubes, and viable and non-viable cells were counted using an automated Cell Counter (Cell Counter Model R1; Olympus, Tokyo, Japan). Cell viability was calculated as the percentage of viable cells relative to the total number of cells counted.

### 4.8. Superoxide Anion Measurement Assay of dHL-60 Cells

Superoxide anion production was quantified using a WST-1 reduction assay with 1-methoxy PMS as an electron mediator [25]. RA-differentiated HL-60 cells were harvested, washed, and resuspended in HBSS (pH 7.4) at a density of 1 × 10^6^ cells/mL. Cells were seeded in 96-well plates with WST-1 (50 mM), 1-methoxy PMS (1 mM final concentration), and 10 nM PMA or vehicle control. The plates were incubated at 37 °C for 30 min. The absorbance of the generated formazan dye was measured at 440 nm with a reference wavelength of 600 nm using a microplate reader. Superoxide anion production was calculated from the difference in absorbance between stimulated and unstimulated cells, and the specificity was confirmed by inhibition with superoxide dismutase.

### 4.9. Quantification of Extracellular DNA

Differentiated HL-60 (dHL-60) cells were treated with or without hispidin for 24 h and seeded at a density of 1 × 10^6^ cells/mL in 96-well plates. After stimulation with 10 μM A23187 or 1 nM PMA for 3 h, the cells were treated with 20 U/mL micrococcal nuclease (MNase; New England Biolabs Japan, Tokyo, Japan) for 20 min at 37 °C. Supernatants containing extracellular DNA were collected after centrifugation at 200× *g* for 8 min at 4 °C. Extracellular DNA was quantified using SYTOX Green (Thermo Fisher Scientific, Waltham, MA, USA) [26].

### 4.10. Total Cell Protein Extraction

Total cell protein was extracted as previously described [27]. Cells were gently suspended in lysis buffer containing 20 mM HEPES-NaOH (pH 7.8), 15 mM KCl, 2 mM MgCl2, 0.5% NP-40, and a protease inhibitor cocktail. After centrifugation at 15,000× *g* for 20 min, the supernatant was collected, and the protein concentration was determined using a BCA protein assay kit (Pierce BCA Protein Assay Kit; Thermo Fisher Scientific).

### 4.11. Western Blotting

Total cell protein extracts (20 μg) were mixed with a sample buffer containing 62.5 mM Tris-HCl (pH 6.8), 2% SDS, 5% glycerol, 0.8% 2-mercaptoethanol, and 0.012% bromophenol blue. Samples were boiled for 5 min and separated by SDS-PAGE on 12.5% polyacrylamide gels. Proteins were then transferred to PVDF membranes. Membranes were blocked with 5% skim milk in TBST containing 20 mM Tris-HCl (pH 7.5), 0.15 M NaCl, and 0.05% Tween 20, and then incubated with anti-PAD4 primary antibody (Abcam, ab50332, 1:1000). After washing, the membranes were incubated with an anti-rabbit secondary antibody (1:3000). Signals were detected using ImmunoStar Zeta (Wako Pure Chemical Industries, Osaka, Japan) and visualized using Amersham Imager 600 (GE Healthcare Life Sciences, Tokyo, Japan).

### 4.12. Statistical Analysis

All data are presented as mean ± SD unless otherwise stated. Animal experiments were performed with six animals per group. For continuous variables involving comparisons of multiple treatment groups against the UVB + vehicle group, one-way ANOVA followed by Dunnett’s multiple comparison test was performed. For ordinal data obtained from the modified Draize scoring system, the Kruskal–Wallis test followed by Dunn’s multiple comparison test was used. For cell viability analyses, to compare each hispidin concentration with the 0 μM control, Holm-adjusted Welch *t*-tests were used. For other two-group comparisons in the in vitro assays, Student’s *t*-test was used, where appropriate. Statistical analyses were performed using the SPSS software (version 20) (IBM Corp., Armonk, NY, USA). Statistical significance was set at *p* < 0.05.

## 5. Conclusions

In conclusion, topical hispidin ameliorated acute UVB-induced skin inflammation by reducing oxidative stress, decreasing neutrophil infiltration, and suppressing NET-related inflammatory responses. These findings support the view that hispidin acts as a topical modulator of oxidative stress- and NET-associated skin inflammation. Additional studies are required to define its precise molecular targets, compare its efficacy with benchmark compounds, and validate its effects in more clinically relevant experimental systems, including primary human neutrophils and models that better reflect the inflammatory microenvironment of UVB-damaged skin.

## Data Availability

The data supporting the findings of this study are available within the article and its Supplementary Materials. High-resolution original images and raw quantification data supporting the main findings are provided as Supplementary Materials/Source Data. Additional information is available from the corresponding author upon reasonable request.

## Supplementary Materials

The following supporting information can be downloaded at: https://www.mdpi.com/article/10.3390/ijms27083667/s1.

## Abbreviations

The following abbreviations are used in this manuscript:

citH3	Citrullinated histone H3
NET	Neutrophil extracellular trap
NETosis	Neutrophil extracellular trap formation
PMA	Phorbol myristate acetate
RA	Retinoic acid
ROS	Reactive oxygen species
UV	Ultraviolet

## References

[1] Byrne S.N., Beaugie C., O’Sullivan C., Leighton S., Halliday G.M. (2011). The immune-modulating cytokine and endogenous Alarmin interleukin-33 is upregulated in skin exposed to inflammatory UVB radiation. Am. J. Pathol..

[2] Gag O., Dinu S., Manea H., Marcovici I., Pinzaru I., Popovici R., Crainiceanu Z., Gyori Z., Iovanescu G., Chiriac S. (2023). UVA/UVB Irradiation Exerts a Distinct Phototoxic Effect on Human Keratinocytes Compared to Human Malignant Melanoma Cells. Life.

[3] Arndt S., Unger P., Ivanova I., Baumler W., Drexler K., Berneburg M., Karrer S. (2025). Cold Atmospheric Plasma Improves the Therapeutic Success of Photodynamic Therapy on UV-B-Induced Squamous Cell Carcinoma in Hairless Mice. Pharmaceuticals.

[4] Sherwani M.A., Burns E.M., Ahmad I., Jasser A.O., Chandra A., Yusuf N. (2024). Tualang Honey Has a Protective Effect Against Photodamage and Skin Cancer: An In Vivo Study. Nutrients.

[5] Brinkmann V., Reichard U., Goosmann C., Fauler B., Uhlemann Y., Weiss D.S., Weinrauch Y., Zychlinsky A. (2004). Neutrophil extracellular traps kill bacteria. Science.

[6] Papayannopoulos V. (2018). Neutrophil extracellular traps in immunity and disease. Nat. Rev. Immunol..

[7] Inaba I., Hiramoto K., Yamate Y., Morita A., Tsutsumi T., Yasuda H., Sato E.F. (2024). Inhibiting Neutrophil Extracellular Traps Protects against Ultraviolet B-Induced Skin Damage: Effects of Hochu-ekki-to and DNase I. Int. J. Mol. Sci..

[8] Azzouz D., Palaniyar N. (2024). How Do ROS Induce NETosis? Oxidative DNA Damage, DNA Repair, and Chromatin Decondensation. Biomolecules.

[9] Zhou Y., An L.L., Chaerkady R., Mittereder N., Clarke L., Cohen T.S., Chen B., Hess S., Sims G.P., Mustelin T. (2018). Evidence for a direct link between PAD4-mediated citrullination and the oxidative burst in human neutrophils. Sci. Rep..

[10] Zambrano F., Uribe P., Schulz M., Hermosilla C., Taubert A., Sanchez R. (2025). Antioxidants as Modulators of NETosis: Mechanisms, Evidence, and Therapeutic Potential. Int. J. Mol. Sci..

[11] Nishidono Y., Tanaka K. (2024). Phytochemicals of Alpinia zerumbet: A Review. Molecules.

[12] Palkina K.A., Ipatova D.A., Shakhova E.S., Balakireva A.V., Markina N.M. (2021). Therapeutic Potential of Hispidin-Fungal and Plant Polyketide. J. Fungi.

[13] Manda-Handzlik A., Bystrzycka W., Wachowska M., Sieczkowska S., Stelmaszczyk-Emmel A., Demkow U., Ciepiela O. (2018). The influence of agents differentiating HL-60 cells toward granulocyte-like cells on their ability to release neutrophil extracellular traps. Immunol. Cell Biol..

[14] Meagher L.C., Cotter T.G. (1988). The degranulation response in differentiated HL-60 cells. Clin. Exp. Immunol..

[15] Rincón E., Rocha-Gregg B.L., Collins S.R. (2018). A map of gene expression in neutrophil-like cell lines. BMC Genom..

[16] Ke J., Yan Y. (2024). Allicin attenuates UVB-induced photodamage of keratinocytes by inhibiting NLRP3 inflammasomes and activating the PI3K/Akt pathway. Arch. Dermatol. Res..

[17] Hasegawa T., Noguchi S., Nakashima M., Miyai M., Goto M., Matsumoto Y., Torii S., Honda S., Shimizu S. (2024). Alternative autophagy dampens UVB-induced NLRP3 inflammasome activation in human keratinocytes. J. Biol. Chem..

[18] Hiramoto K., Kobayashi H., Yamate Y., Ishii M., Sato E.F. (2012). Intercellular pathway through hyaluronic acid in UVB-induced inflammation. Exp. Dermatol..

[19] Hiramoto K., Yamate Y., Yokoyama S. (2018). Ultraviolet B eye irradiation aggravates atopic dermatitis via adrenocorticotropic hormone and NLRP3 inflammasome in NC/Nga mice. Photodermatol. Photoimmunol. Photomed..

[20] Draize J.H., Woodard G., Calvery H.O. (1944). Methods for the study of irritation and toxicity of substances applied topically to the skin and mucous membranes. J. Pharmacol. Exp. Ther..

[21] Titford M. (2005). The long history of hematoxylin. Biotech. Histochem..

[22] Zauat S., Becker L.L., Kaindl A.M. (2020). Immunofluorescence Staining of Paraffin sections step by step. Front. Neuroanat..

[23] Collins S.J., Ruscetti F.W., Gallagher R.E., Gallo R.C. (1978). Terminal differentiation of human promyelocytic leukemia cells induced by dimethyl sulfoxide and other polar compounds. Proc. Natl. Acad. Sci. USA.

[24] Bunce C.M., Fisher A.G., Toksoz D., Brown G. (1983). Isolation and characterisation of dimethylsulphoxide resistant variants from the human promyeloid cell line HL60. Exp. Hematol..

[25] Tan A.S., Berridge M.V. (2000). Superoxide produced by activated neutrophils efficiently reduces the tetrazolium salt, WST-1 to produce a soluble formazan: A simple colorimetric assay for measuring respiratory burst activation and for screening anti-inflammatory agents. J. Immunol. Methods.

[26] Ohinata H., Obama T., Makiyama T., Watanabe Y., Itabe H. (2022). High-density lipoprotein suppresses neutrophil extracellular traps enhanced by oxidized low-density lipoprotein or oxidized phospholipids. Int. J. Mol. Sci..

[27] Schreiber E., Matthias P., Muller M.M., Schaffner W. (1989). Rapid detection of octamer binding proteins with ‘mini extracts’, prepared from a small number of cells. Nucleic Acids Res..

