# Supplementary material for: Hispidin Ameliorates Acute Ultraviolet B-Induced Skin Inflammation by Targeting Reactive Oxygen Species-Dependent Neutrophil Extracellular Trap Formation
Source: Int J Mol Sci. 2026 Apr 20;27(8):3667. doi: 10.3390/ijms27083667 (PMC13115934; doi:10.3390/ijms27083667)

Figure S1. Effects of hispidin on dHL-60 cell viability after 48 h treatment. Differentiated HL-60 cells were treated with the indicated concentrations of hispidin for 48 h. Bars show mean ± SD and dots indicate independent replicates (n = 3). Statistical significance was analyzed using Holm-adjusted Welch t-tests versus the 0 μM control.

Figure S1.

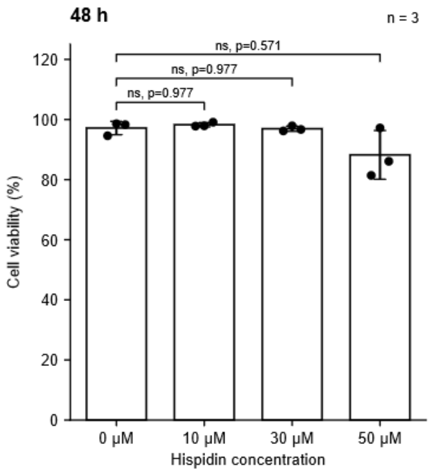

Supplement: Supplementary file 1 [file ijms-27-03667-s001.zip › ijms-4235806-supplementary.pdf]
